# Motor subtypes and clinical characteristics in sporadic and genetic Parkinson's disease groups: analysis of the PPMI cohort

**DOI:** 10.3389/fneur.2023.1276251

**Published:** 2023-10-25

**Authors:** Eun Hye Jeong, Jae Yong Lee, Sun-Ku Han, Yoo Sung Song

**Affiliations:** ^1^Department of Neurology, Bundang Jesaeng General Hospital, Seongnam-si, Gyeonggi-do, Republic of Korea; ^2^Department of Nuclear Medicine, Seoul National University Bundang Hospital, Seongnam-si, Gyeonggi-do, Republic of Korea; ^3^College of Medicine, Seoul National University, Seoul, Republic of Korea

**Keywords:** *GBA*, I-123 FP-CIT SPECT, *LRRK2*, Parkinson's disease, PPMI, sporadic PD

## Abstract

**Introduction:**

The extensive clinical variations observed in Parkinson's disease (PD) pose challenges in early diagnosis and treatment initiation. However, genetic research in PD has significantly transformed the clinical approach to its treatment. Moreover, researchers have adopted a subtyping strategy based on homogeneous clinical symptoms to improve clinical diagnosis and treatment approaches. We conducted a study to explore clinical characteristics in genetic PD groups with motor symptom subtyping.

**Methods:**

Data was driven from the Parkinson's Progression Markers Initiative (PPMI) database. The sporadic PD (sPD) group and the genetic PD group including patients with leucine-rich kinase 2 (*LRRK2*) or glucosylceramidase β (*GBA*) mutations were analyzed. Motor subtyping was performed using Movement Disorder Society-Unified Parkinson's disease rating scale (MDS-UPDRS) scores. I-123 FP-CIT SPECT scans were used to calculate specific binding ratios (SBRs) in the caudate and putamen. Clinical symptoms of each group were also compared.

**Results:**

MDS-UPDRS III scores were lower in the *LRRK2* group, compared with the *GBA* and sPD group (*P* < 0.001), but no significant differences in striatal SBRs. The putaminal SBR value of the *LRRK2* group was higher than the sPD group (*P* < 0.05). Within the *GBA* group, we observed lower SBR values in the postural instability/gait difficulty (PIGD) subtype *GBA* group compared to the tremor-dominant (TD) subtype *GBA* group (*P* < 0.05). The TD subtype *GBA* group exhibited superior putaminal SBRs compared to the TD subtype sPD group (*P* < 0.05). The TD subtype *LRRK2* group had better putaminal SBR values (*P* < 0.001) and MDS-UPDRS Part III scores (*P* < 0.05) compared to the TD sPD group.

**Discussions:**

Our subtyping approach offers valuable insights into the clinical characteristics and progression of different genetic PD subtypes. To further validate and expand these findings, future research with larger groups and long-term follow-up data is needed. The subtyping strategy based on motor symptoms holds promise in enhancing the diagnosis and treatment of genetic PD.

## Introduction

Parkinson's disease (PD) is a degenerative neurological condition that primarily affects the motor system as a result of a loss of dopamine in the nigrostriatal system (1). While there are variations among different ethnic groups, ~10–15% of PD cases have a familial history and follow a Mendelian inheritance pattern, whereas the remaining cases are considered sporadic (2). Among the various gene mutations that have been confirmed, the most common causes of autosomal dominant PD are mutations in leucine-rich kinase 2 (*LRRK2*) and mutations in glucosylceramidase β (*GBA*) (3). Both of these mutations are closely associated with the development of late-onset typical PD, which is characterized by the presence of Lewy bodies, a hallmark of the disease. Over the past 2–3 decades since the initial reports on *GBA* and *LRRK2* PD (4, 5), numerous clinical observations have highlighted differences in clinical manifestations compared to sporadic PD (sPD). *GBA* PD is known to be linked with reduced survival, rapid disease progression, and more severe motor and cognitive impairment (6). In contrast, *LRRK2* PD is associated with a slower progression of motor symptoms and a lower risk of cognitive dysfunction (7).

Given the complex range of symptoms associated with PD, clinicians have proposed a classification system based on motor symptoms, specifically distinguishing between tremor-dominant (TD) and postural instability/gait difficulty (PIGD) subtypes (8). Identifying these subtypes is relevant for understanding disease progression, cognitive decline, and autonomic dysfunction (9–11). Consequently, determining the subtype of PD in individual patients has garnered interest due to its potential for predicting prognosis. However, there is a lack of significant studies exploring the clinical subtypes within the genetic PD population, primarily due to limited groups of PD patients and the necessity for genetic screening. It is worth noting that variants of *LRRK2* and *GBA* are also implicated in sporadic PD cases, highlighting the heterogeneous nature of the disease. Therefore, in addition to genetic factors, a phenotype-based analysis is essential, particularly for personalized therapy and prognosis prediction.

The Parkinson's Progression Markers Initiative (PPMI) repository is a collaborative effort involving multiple centers and contains comprehensive longitudinal clinical, imaging, and biological data from patients with PD. This repository is regularly updated to provide ongoing follow-up information. Notably, the group within this repository encompasses not only individuals with sPD but also those with genetic PD who have *LRRK2* or *GBA* mutations.

The objective of our study was to assess the motor subtypes present in different genetic PD groups and compare them to the sPD group. We hypothesized that there would be variations in the [I-123] N-ω-fluoropropyl-2β-carbomethoxy-3β-(4-iodophenyl) nortropane (I-123 FP-CIT) single positron emission computed tomography (SPECT) findings based on the different genetic PD subtypes. Furthermore, we anticipated that these differences would serve as clinical indicators of the current condition and prognosis for individual patients.

## Methods

### Patients

Clinical data from both the sporadic PD (sPD) and genetic PD groups were obtained from the PPMI database (http://www.ppmi-info.org) in February 2022. Inclusion criteria for the sPD patients were as follows: individuals with clinically significant movement disorders, Hoehn and Yahr (H&Y) stage I or II at the beginning of the study, aged 30 years or older, and exhibiting dopamine transporter (DAT) deficits on baseline I-123 FP-CIT SPECT images. The inclusion criteria for genetic PD patients were as follows: individuals with clinically relevant movement disorders, diagnosed with PD for 7 years or less at the screening, H&Y stage I, II, or III at baseline, aged 18 years or older, and showing DAT deficits on baseline I-123 FP-CIT SPECT images, with confirmed mutations in *LRRK2* or *GBA*. Due to the varying inclusion criteria for the sPD and genetic PD groups, the analysis was based on the 2-year follow-up data of the sPD group (12). sPD patients without 2-year follow-up I-123 FP-CIT SPECT images were excluded from the study. We evaluated the corresponding H&Y stages, Movement Disorder Society-Unified Parkinson's disease rating scale (MDS-UPDRS) scores, Scale for Outcomes in Parkinson's disease-Autonomic (SCOPA-AUT) scores, and Montreal Cognitive Assessment (MoCA) scores. To subtype patients based on motor symptoms, we calculated the ratio of the mean tremor scores to the mean PIGD scores from the MDS-UPDRS scores. TD patients were defined as having a ratio of ≥1.15, PIGD patients as having a ratio of ≤ 0.90, and indeterminate patients as having ratios between 0.90 and 1.15 (8). The PPMI trial is performed in accordance with the relevant regulations and guidelines. Written informed consent were obtained from all participants, and subjects were given written informed consent. PPMI was approved, respectively, by all participating institutions' local institutional review boards. All study procedures were conducted in accordance with the 1964 Declaration of Helsinki and its later amendments.

### I-123 FP-CIT SPECT scans

I-123 FP-CIT SPECT scans were conducted ~4 ± 0.5 h after the injection of I-123 FP-CIT (111–185 MBq). The images were reconstructed iteratively, without filtering application. To ensure quality control, the core imaging laboratory of the PPMI conducted technical setup visits prior to study enrollment, performed phantom studies, validated the acquisition protocol, and standardized image acquisition procedures. Analysis of the images were done with a the PMOD software (PMOD Technologies, Zurich, Switzerland), and specific binding ratios [SBRs, (target region/reference region)-1] were calculated for each dominant caudate and putamen, with the occipital cortex serving as the reference tissue. The dominant hemisphere was defined as the side with more severe motor related symptoms. Asymmetry index of the right and left hemispheres were calculated according to a previously published method: |SBRleft–SBRright|/SBR values of age-matched normal controls × 100 (13).

### Statistical analysis

A dedicated statistical software (MedCalc Software, version 20.118, Belgium) was used for analysis. We compared the clinical characteristics of the PD groups and clinical subtypes using one-way ANOVA with Scheffé test for *post-hoc* analysis for parametric factors, and Kruskal-Wallis test with Conover test for *post-hoc* analysis for non-parametric factors. Chi-square test was used to analyze the distribution of clinical subtypes of PD groups. A *P*-value <0.05 was considered to be statistically significant.

## Results

### Baseline characteristics

A total of 239 patients with sporadic PD (sPD) and 187 patients with genetic PD were included in the analysis. Within the genetic PD group, there were 61 patients with *GBA*-associated PD (*GBA* PD) and 126 patients with *LRRK2*-associated PD (*LRRK2* PD). The duration of symptoms did not significantly differ between the sPD and genetic PD patients (59.0 ± 56.7 months vs. 46.4 ± 17.2 months, *P* = 0.07). Additionally, there were no significant differences in terms of age, age at PD onset, Hoehn and Yahr (H&Y) stages, Movement Disorder Society-Unified Parkinson's disease rating scale (MDS-UPDRS) Part II scores, Scale for Outcomes in Parkinson's disease-Autonomic (SCOPA-AUT) scores, and Montreal Cognitive Assessment (MoCA) scores among the sPD, *GBA* PD, and *LRRK2* PD patients (Table 1). The male gender was predominant in the sPD group (*P* < 0.05). Notably, the MDS-UPDRS Part III score was significantly lower in the *LRRK2* PD group compared to both the sPD and *GBA* PD groups (*P* < 0.001). The racial composition of the patients included in our study is as follows: Hispanic/Latino (9.1%), American Indian/Alaska native (0.8%), Asian (1.3%), Black/African American (0.8%), White (87.8%). There were no significant differences of demographic characteristics according to race.

**Table 1:** Baseline characteristics of the *GBA* PD, *LRRK2* PD, and sPD group.

	***GBA* PD (*n* = 61)**	***LRRK2* PD (*n* = 126)**	**sPD (*n* = 239)**	***P*-value**
Age	64.2 ± 8.7	63.3 ± 9.1	63.3 ± 9.4	0.76
Age at PD onset	59.9 ± 9.5	58.2 ± 9.8	59.4 ± 9.6	0.45
Gender (male: female)	34: 27	63: 63	172: 92	<0.05
H&Y staging	1.87 ± 0.50	1.76 ± 0.57	1.78 ± 0.56	0.38
MDS-UPDRS part II score	9.9 ± 6.0	8.5 ± 6.1	8.7 ± 5.1	0.15
MDS-UPDRS part III score	28.3 ± 10.6^a)^	21.9 ± 11.2^b)^	26.1 ± 11.0^a)^	<0.001
SCOPA-AUT	13.7 ± 7.3	12.3 ± 8.2	12.3 ± 7.0	0.20
MoCA score	26.0 ± 2.6	25.8 ± 3.9	26.6 ± 2.6	0.13
Dominant side caudate SBR	1.67 ± 0.74	1.71 ± 0.53^a)^	1.55 ± 0.50^b)^	<0.05
Dominant side putaminal SBR	0.69 ± 0.46	0.69 ± 0.34^a)^	0.57 ± 0.21^b)^	<0.01
Dominant side caudate asymmetry index	11.3 ± 7.7	11.0 ± 9.2	10.6 ± 7.3	0.77
Dominant side putamen asymmetry index	9.9 ± 9.0	10.7 ± 8.0	10.4 ± 8.5	0.47

For a particular variable, values with different superscripts indicate statistically significant difference.

We compared the SBRs and asymmetry indices between the sPD, *GBA* PD, and *LRRK2* PD patients (Table 1). The SBRs of the caudate and putamen in the sPD group were significantly lower than those in the *LRRK2* PD group (*P* < 0.05 and *P* < 0.01, respectively). There were no significant differences of the SBRs of the caudate and putamen in the sPD group and the *GBA* PD group. However, no significant differences were observed in the asymmetry indices of the dominant-side caudate and putamen among any of the groups.

### Clinical subtype analysis

The distribution of clinical subtypes between the sPD, *GBA* PD, and *LRRK2* groups were compared (Table 2). Chi-squared test revealed a significant difference of the distribution of TD, indeterminant, and PIGD subtypes (*P* < 0.01).

**Table 2:** Distribution of clinical subtypes of the sPD, *GBA* PD, and *LRRK2* groups.

	***GBA* PD**	***LRRK2* PD**	**sPD**
TD (%)	28 (47.4)	65 (54.2)	166 (69.5)
Indeterminant (%)	5 (8.4)	10 (8.3)	19 (7.9)
PIGD (%)	26 (44.1)	45 (37.4)	54 (22.6)
Total	59 (100.0)	120 (100.0)	240 (100.0)

We compared the SBRs and asymmetry indices of the PD groups based on the motor subtypes (Table 3). Within the TD subtype, patients in the sPD group exhibited significantly lower caudate SBR values compared to the *LRRK2* PD group (*p* < 0.05). Additionally, they had lower putaminal SBR values compared to both the *GBA* PD and *LRRK2* PD groups (*p* < 0.001). No significant differences were observed in the asymmetry indices of the TD subtype based on the genetics of PD. Regarding the PIGD subtype, there were no significant differences in either the SBR values or asymmetry indices among the different genetic types of PD.

**Table 3:** Clinical characteristics of the PD groups based on the motor subtypes.

	***GBA* PD**	***LRRK2* PD**	**sPD**	***P*-value**
**TD**				
Dominant side caudate SBR	1.83 ± 0.76	1.78 ± 0.53^a)^	1.60 ± 0.51^b)^	<0.05
Dominant side putaminal SBR	0.85 ± 0.52^a)^	0.74 ± 0.37^a)^	0.59 ± 0.20^b)^	<0.001
Dominant side caudate asymmetry index	11.5 ± 7.9	11.4 ± 8.4	10.3 ± 7.1	0.67
Dominant side putamen asymmetry index	10.5 ± 8.9	11.6 ± 8.5	10.2 ± 8.4	0.25
H&Y staging	1.78 ± 0.50	1.65 ± 0.54	1.73 ± 0.48	0.43
MDS-UPDRS part II score	8.46 ± 5.49	7.23 ± 5.06	7.83 ± 4.57	0.38
MDS-UPDRS part III score	27.4 ± 10.7^a)^	22.4 ± 11.7^b)^	25.8 ± 10.2^a)^	<0.05
SCOPA-AUT	13.1 ± 6.2	10.3 ± 6.7	11.8 ± 6.5	0.05
MoCA score	26.1 ± 2.4	25.6 ± 4.3	26.7 ± 2.5	0.18
**PIGD**				
Dominant side caudate SBR	1.48 ± 0.71	1.61 ± 0.53	1.40 ± 0.38	0.26
Dominant side putaminal SBR	0.56 ± 0.38	0.66 ± 0.33	0.53 ± 0.20	0.09
Dominant side caudate asymmetry index	11.4 ± 8.1	11.8 ± 10.8	11.9 ± 8.2	0.97
Dominant side putamen asymmetry index	8.3 ± 8.6	8.8 ± 7.8	10.8 ± 10.5	0.42
H&Y staging	1.92 ± 0.56	1.91 ± 0.60	2.02 ± 0.71	0.86
MDS-UPDRS part II score	11.3 ± 6.6	10.4 ± 7.5	10.9 ± 5.8	0.60
MDS-UPDRS part III score	28.5 ± 11.1^a)^	21.8 ± 10.4^b)^	26.6 ± 13.3	<0.05
SCOPA-AUT	14.2 ± 8.6	15.2 ± 9.8	14.2 ± 7.7	0.97
MoCA score	25.8 ± 2.8	25.8 ± 3.8	26.1 ± 2.7	0.91

For a particular variable, values with different superscripts indicate statistically significant difference.

The symptoms of the PD groups were compared based on the motor subtypes (Table 3). For the TD subtypes, the MDS-UPDRS Part III score was significantly lower in the *LRRK2* PD group compared to both the sPD and *GBA* PD groups (*P* < 0.05). For the PIGD subtypes, the MDS-UPDRS Part III score was significantly lower in the *LRRK2* PD group compared to only the *GBA* PD group (*P* < 0.05).

SBR values and asymmetry indices of motor subtypes were compared according to PD groups (Table 4). In the *GBA* PD group, the TD subtype had a significantly higher putminal SBR value compared with the PIGD subtype (*P* < 0.05). In the *LRRK2* PD group, the PIGD subtype had significantly lower putamen asymmetry index compared with the TD and indeterminant subtypes (*P* < 0.05). In the sPD group, the TD subtype had a significantly higher putminal SBR value compared with the indeterminant subtype (*P* < 0.05).

**Table 4:** Clinical characteristics of motor subtypes according to PD groups.

	**TD**	**Indeterminant**	**PIGD**	***P*-value**
***GBA*** **PD**				
Dominant side caudate SBR	1.83 ± 0.76	1.82 ± 0.80	0.48 ± 0.71	0.22
Dominant side putaminal SBR	0.85 ± 0.52^a)^	0.57 ± 0.27	0.56 ± 0.38^b)^	<0.05
Dominant side caudate asymmetry index	11.5 ± 7.9	13.2 ± 5.0	11.4 ± 8.1	0.73
Dominant side putamen asymmetry index	10.5 ± 8.9	15.7 ± 12.2	8.3 ± 8.6	0.27
H&Y staging	1.78 ± 0.50	2.00 ± 0.00	1.92 ± 0.56	0.50
MDS-UPDRS part II score	8.5 ± 5.5	9.0 ± 0.0	11.3 ± 6.6	0.28
MDS-UPDRS part III score	21.8 ± 10.3	28.8 ± 10.3	22.4 ± 10.0	0.39
SCOPA-AUT	13.1 ± 6.2	13.6 ± 8.7	14.2 ± 8.6	0.94
MoCA score	26.1 ± 2.4	25.6 ± 3.6	25.8 ± 2.8	0.97
***LRRK2*** **PD**				
Dominant side caudate SBR	1.78 ± 0.53	1.55 ± 0.58	1.61 ± 0.53	0.21
Dominant side putaminal SBR	0.74 ± 0.37	0.53 ± 0.25	0.66 ± 0.33	0.06
Dominant side caudate asymmetry index	11.4 ± 8.4	8.4 ± 6.6	11.8 ± 10.8	0.60
Dominant side putamen asymmetry index	11.6 ± 8.5^a)^	13.1 ± 7.1^a)^	8.8 ± 7.8^b)^	<0.05
H&Y staging	1.65 ± 0.54	1.60 ± 0.52	1.91 ± 0.60	<0.05
MDS-UPDRS part II score	7.2 ± 5.1^a)^	7.9 ± 4.3	10.4 ± 7.5^b)^	<0.05
MDS-UPDRS part III score	22.4 ± 11.7	19.1 ± 12.0	21.8 ± 10.4	0.63
SCOPA-AUT	10.3 ± 6.7^a)^	12.4 ± 8.2	15.2 ± 9.8^b)^	<0.05
MoCA score	25.6 ± 4.3	26.3 ± 2.6	25.8 ± 3.8	0.95
**sPD**				
Dominant side caudate SBR	1.60 ± 0.51	1.50 ± 0.46	1.40 ± 0.38	0.07
Dominant side putaminal SBR	0.59 ± 0.20^a)^	0.48 ± 0.15^b)^	0.53 ± 0.20	<0.05
Dominant side caudate asymmetry index	10.3 ± 7.1	8.7 ± 6.9	11.9 ± 8.2	0.31
Dominant side putamen asymmetry index	10.2 ± 8.4	10.4 ± 6.4	10.8 ± 10.5	0.78
H&Y staging	1.73 ± 0.48^a)^	1.74 ± 0.56	2.02 ± 0.71^b)^	<0.05
MDS-UPDRS part II score	7.8 ± 4.6^a)^	9.7 ± 4.6	10.9 ± 5.8^b)^	<0.01
MDS-UPDRS part III score	25.8 ± 10.2	27.3 ± 10.4	26.6 ± 13.3	0.86
SCOPA-AUT	11.8 ± 6.5	13.2 ± 9.2	14.2 ± 7.7	0.19
MoCA score	26.7 ± 2.5	27.0 ± 3.2	26.1 ± 2.7	0.20

For a particular variable, values with different superscripts indicate statistically significant difference.

Symptoms of motor subtypes were compared according to PD groups (Table 4). There were no significant differences of symptoms between the motor subtypes of the *GBA* PD group. The MDS-UPDRS Part II score and SCOPA-AUT of the PIGD subtype was higher than the TD subtype in the *LRRK2* group (*P* < 0.05). The H&Y staging and MDS-UPDRS Part II score of the PIGD subtype was higher than the TD subtype in the sPD group (*P* < 0.05, *P* < 0.01, respectively).

## Discussions

Genetic research in Parkinson's disease (PD) has contributed to a novel clinical approach for its treatment. The extensive clinical variations observed in PD pose a significant challenge in terms of early diagnosis and treatment initiation. Recent investigations into motor symptoms, non-motor symptoms, disease progression patterns, and imaging characteristics have aided in revealing the unique features and prognosis of genetic PD. However, due to the relatively smaller proportion of genetic PD cases compared to sporadic PD, randomized controlled studies involving a large number of individuals with genetic PD are rare. To address this limitation, researchers have pursued subtyping PD based on homogeneous clinical symptoms, aiming to improve the accuracy of clinical diagnosis and treatment strategies (14).

In our study, we found no significant differences in the MDS-UPDRS III scores between the *GBA* group and the sPD group. Also, there were no significant differences in the striatal SBRs between these two groups. These results differ from a previous study conducted by Brockmann et al. (6) which implicated a more rapid disease progression in the *GBA* group compared with the sPD group. The differences between the two studies could be attributed to variations in the number of subjects and the composition of the groups. In our study, we included both the *GBA* and *LRRK2* groups. Additionally, discrepancies may arise from the differences in baseline characteristics and inclusion criteria for genetic and sPD groups within the PPMI. In our analysis, we specifically focused on the 2-year data of the sPD group, while Brockmann et al. observed prominent differences in motor symptoms between the two groups during a 3-year observation period. Furthermore, their study had a much smaller sample size (39 patients) compared to our present study (300 patients). Given the lack of significant differences in disease duration between the two groups in our study, further follow-up data may be necessary for a more comprehensive understanding and clarification of these findings.

Our analysis of subtypes within the groups has provided valuable insights into disease progression. In the *GBA* group, we observed lower SBR values in the PIGD subtype *GBA* group compared to the TD subtype *GBA* group. However, there were no significant differences in the MDS-UPDRS III scores between these two groups. Interestingly, it appears that the *GBA* PD group has a higher proportion of PIGD subtypes and a lower proportion of TD subtypes compared to the sPD group. Specifically, a previous study reported that a specific E226K *GBA* mutation is linked to a swifter progression of postural and gait instability disorders, but not tremor (15). This may explain the higher prevalence of PIGD subtypes in *GBA* PD cases.

The TD subtype *GBA* group exhibited superior putaminal SBRs compared to the TD subtype sPD group; however, the MDS-UPDRS III scores of these two groups did not show any significant differences. This finding suggests that factors other than putamen degeneration might play a role in contributing to the motor symptoms observed in the TD subtype *GBA* group. In PD, the severity of tremor has been reported not to correlate with dopaminergic degeneration of the striatum (16). Instead, it has been suggested that increased neural firing in the basal ganglia leads to heightened excitability in the primary motor cortex (17). *GBA* protein is widely present in various body organs, including the brain, and it exhibits a higher expression level than *LRRK2* (www.proteinatlas.org). Given its ubiquity, *GBA* mutations may impact sites beyond the striatum, potentially contributing to tremor-related symptoms through different mechanisms. Further investigations, such as functional connectivity studies, may help to elucidate the underlying pathophysiology associated with *GBA* mutations and their impact on motor symptoms in PD.

Furthermore, when comparing the *LRRK2* group and the *GBA* group, we found no significant differences in the SBRs; however, the MDS-UPDRS III scores were higher in the *GBA* group. This trend was consistent when considering both TD and PIGD subtypes within each group. Additionally, in our study, we observed better SBRs in the *LRRK2* group compared to the sPD group, while the MDS-UPDRS III scores were higher in the sPD group than in the *LRRK2* group. Subtype analysis revealed that the striatal SBRs of the TD subtype *LRRK2* group were superior to those of the TD subtype sPD group, while no significant differences were observed in the striatal SBRs between the PIGD subtype *LRRK2* and PIGD subtype sPD groups. Similarly, the MDS-UPDRS III scores of the TD subtype *LRRK2* group indicated less disease progression compared to the TD subtype sPD group, whereas no significant differences were found in the MDS-UPDRS III scores between the PIGD subtype *LRRK2* and PIGD subtype sPD groups. Consequently, the significant differences observed in the striatal SBRs between the entire *LRRK2* and sPD groups can likely be attributed to the TD subtypes. These findings suggest that *LRRK2* mutations have a lesser impact on the pathophysiology of tremor-related symptoms.

In our study, we observed differences in the putamen asymmetry index between the TD and PIGD subtypes only in *LRRK2* PD group. However, there were no significant differences in the asymmetry index between TD and PIGD subtypes in the *GBA* PD and sPD groups. A prior investigation into the progression of striatal denervation and asymmetry based on motor subtypes provides noteworthy insights (13). In this study, TD subtypes exhibited the largest asymmetry index at baseline, which gradually decreases over a 4-year follow-up period. No significant changes in asymmetry indices were observed during the follow-up period for the PIGD subtype. Furthermore, the asymmetry indices between the TD and PIGD subtypes were most prominently different at the baseline, but this difference became non-significant after the 4-year follow-up. Regarding the slower progression of *LRRK2* PD, our study findings suggest a potential link with the fact that we observed that the asymmetry indices were higher in the TD subtype than in the PIGD subtype only within the *LRRK2* PD group.

The identification of phenotypes in genetic Parkinson's disease (PD) has been limited by the lack of large groups. Subtyping based on TD and PIGD groups has proven useful in evaluating distinct patterns of dopaminergic denervation and disease progression in sPD (11, 18). Despite the presence of various mutations in both the *LRRK2* PD and *GBA* PD groups, patients with different *LRRK2* mutations share remarkably similar clinical characteristics. This similarity is also observed among patients with *GBA* mutations (19, 20). *LRRK2* variants are known to contribute to PD pathology by upregulating *LRRK2* kinase activity, whereas *GBA* variants compromise α-synuclein degradation by causing loss of glucocerebrosidase function (21–23). Our study aimed to demonstrate changes in clinical parameters for each type of genetic PD by evaluating motor symptom characteristics through subtyping. This approach may offer valuable insights into the disease progression of different genetic PD types and contribute to a better understanding of their clinical manifestations.

Our study has some limitations. Firstly, each genetic PD group comprises a heterogeneous number of gene mutations, which may influence the expression of phenotypes. The racial composition of the PPMI database is predominantly White, with 88% representation. However, racial differences were not considered in the data analysis of this study. Secondly, the data were collected from multiple institutions, which could introduce variations in the I-123 FP-CIT SPECT images. However, it is essential to note that the core imaging lab of PPMI conducts quality control of the images to ensure reliability.

In conclusion, our study offers valuable insights into the clinical characteristics and progression of different genetic PD subtypes. However, further research with larger groups and long-term follow-up data is required to validate and expand upon these findings. The subtyping approach based on motor symptoms holds promise as a strategy to enhance the diagnosis and treatment of genetic PD.

## Data availability statement

The data analyzed in this study was obtained from the Parkinson's Progression Markers Initiative (PPMI; https://www.ppmi-info.org/), the following licenses/restrictions apply: Investigators seeking access to PPMI data must sign the Data Use Agreement, submit an Online Application and comply with the study Publications Policy. Requests to access these datasets should be directed to PPMI, https://ida.loni.usc.edu/collaboration/access/appLicense.jsp.

## Ethics statement

The studies involving humans were approved by the respective IRBs of the institutions participating in the PPMI trial (the list of participating institutions is available as a Supplementary Table). The studies were conducted in accordance with the local legislation and institutional requirements. The participants provided their written informed consent to participate in this study.

## Author contributions

EJ: Conceptualization, Data curation, Formal analysis, Investigation, Methodology, Project administration, Writing—original draft, Writing—review and editing. JL: Investigation, Methodology, Writing—review and editing. S-KH: Writing—review and editing. YS: Conceptualization, Data curation, Formal analysis, Funding acquisition, Investigation, Methodology, Resources, Supervision, Validation, Visualization, Writing—original draft, Writing—review and editing.
